# Giant ovarian teratoma: an important differential diagnosis of pelvic masses in children

**DOI:** 10.1590/0100-3984.2016.0026

**Published:** 2017

**Authors:** Felipe Nunes Figueiras, Márcio Luís Duarte, Élcio Roberto Duarte, Daniela Brasil Solorzano, Jael Brasil de Alcântara Ferreira

**Affiliations:** 1 Santa Casa de Santos – Radiologia, Santos, SP, Brazil.; 2 Hospital São Camilo, São Paulo, SP, Brazil.

**Figure 1:**
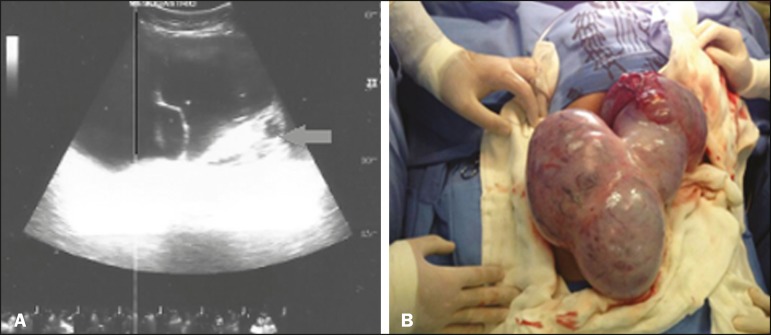
A: Ultrasound of the abdomen, showing a massive solid-cystic formation with a pronounced solid component (arrow). B: Intraoperative photograph showing the large volume of the lesion and its encapsulated appearance.

**Figure 2:**
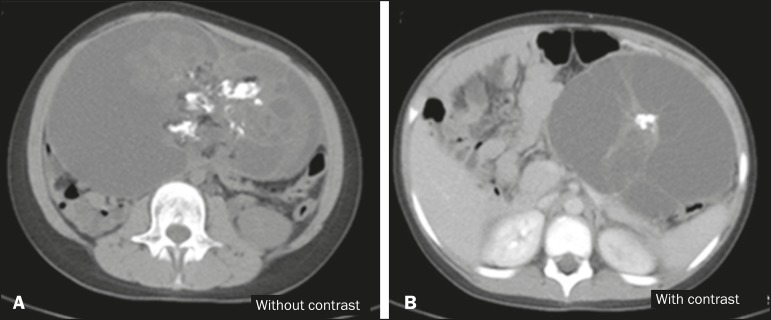
A: Non-contrast-enhanced axial CT scan showing an extensive solid-cystic formation, with a fatty component, a liquid component, and calcifications. B: Intravenous contrast-enhanced axial CT scan showing a compressive effect on and displacement of the structures adjacent to the lesion—the pancreas, abdominal aorta, inferior vena cava, small intestine, and left kidney.

Dear Editor,

An 8-year-old female patient presented with diffuse abdominal pain accompanied by progressive distension. Physical examination revealed a large abdominal mass, predominantly in the mesogastrium, that was depressible and painless on palpation. Ultrasound showed a solid-cystic formation extending from the epigastrium to the hypogastrium, with a calcium component and an air-fluid level (Figure 1). Computed tomography (CT) showed a massive solid-cystic formation, with a fat component and soft tissue, as well as calcifications, measuring 12.6 × 19.2 × 20.8 cm, exerting a significant mass effect, displacing the small intestine, aorta, and inferior vena cava, as well as causing slight compression of the pancreas, kidneys, and ureters, with no apparent signs of infiltration (Figure 2). Intraoperatively, the mass was seen to be adhered to the left fallopian tube and to the greater omentum (Figure 1). The tumor was excised without complications, and the patient was discharged five days later. A follow-up abdominal ultrasound revealed no changes.

The occurrence of an abdominal mass in a child should always be evaluated by a pediatrician. The main differential diagnoses are organomegaly and fecal impaction. When abdominal palpation produces nonspecific findings, further investigation, employing imaging methods, is required^(1)^.

Ovarian teratoma is the most prevalent germ cell neoplasm, accounting for approximately 32% of all ovarian neoplasms, and can be divided into mature or immature teratoma depending on its cellular differentiation^(1)^. The cellular components of this lesion are pronounced and varied, potentially encompassing respiratory epithelium, skin, cartilage, mucosa, and neural epithelium^(2-5)^. It is a benign neoplasm, presenting on physical examination as a palpable pelvic mass, typically 5–10 cm in diameter, and occurs bilaterally in 10–15% of cases^(1)^. In 10% of cases, it is considered an emergency, presenting the typical profile of acute abdomen, due to torsion of the vascular pedicle that occurs secondary to its growth^(6)^. The clinical diagnoses of abdominal masses are diverse and imprecise, requiring complementary diagnostic imaging^(7)^.

Abdominal X-ray is nonspecific for ovarian teratoma and can occasionally show calcifications in the area surrounding the lesion. Ultrasound and CT are the main imaging methods for the detection of this disease, the rapid detection of which demands recognition of the typical imaging patterns, particularly in cases of emergency (acute onset). Although CT also has high specificity and sensitivity, particularly for the detection of cystic teratoma, it is not routinely employed, because it involves the use of ionizing radiation. The combination of various imaging methods is an essential part of the surgical planning^(8)^. The histological study is also of importance, determining the macroscopic and microscopic aspect of the lesion, as well as the prognosis. Surgical treatment—excision of the lesion—is the gold standard^(8)^.
